# Plasma p‐tau217 detects Alzheimer's disease co‐pathology in cerebral amyloid angiopathy: Comparison to CSF biomarkers in the ANGMAR cohort

**DOI:** 10.1002/alz.71709

**Published:** 2026-08-04

**Authors:** Aida Fernández‐Lebrero, Joan Jiménez‐Balado, Greta García‐Escobar, José Contador, Isabel Estraguès‐Gázquez, Laia Peraferrer‐Montesinos, Rosa María Manero‐Borràs, Laura Ludovica Gramegna, Marc Viles, Ana Rodríguez Campello, Paula Ortiz‐Romero, Marina de Diego, Marta del Campo, Javier Torres‐Torronteras, Esther Jiménez‐Moyano, Helena Blasco‐Forniés, Marc Suárez‐Calvet, Angel Ois, Albert Puig‐Pijoan, Irene Navalpotro‐Gómez

**Affiliations:** ^1^ Hospital del Mar Research Institute Barcelona Spain; ^2^ Servei de Neurologia Hospital del Mar Barcelona Spain; ^3^ Department of Medicine and Life Sciences Universitat Pompeu Fabra Barcelona Spain; ^4^ Barcelonaβeta Brain Research Center (BBRC) Pasqual Maragall Foundation Barcelona Spain; ^5^ Neuroradiology Department Hospital del Mar Barcelona Spain

**Keywords:** Alzheimer's disease, amyloid beta, blood biomarker, cerebral amyloid angiopathy, diagnosis, phosphorylated tau 217

## Abstract

**INTRODUCTION:**

Cerebral amyloid angiopathy (CAA) frequently co‐occurs with Alzheimer's disease (AD), generating mixed vascular–neurodegenerative phenotypes. Plasma phosphorylated tau (p‐tau)217 is a robust biomarker of AD, but its performance in CAA remains unclear.

**METHODS:**

We studied 231 participants, including 50 patients with CAA (Boston v2.0), 154 with AD, and 27 cognitively unimpaired controls. Plasma p‐tau217 was measured on an automated platform and compared to cerebrospinal fluid (CSF)–defined AD status. Associations with magnetic resonance imaging (MRI) markers of CAA burden were evaluated.

**RESULTS:**

Among CAA participants, 29 met CSF criteria for AD co‐pathology. Plasma p‐tau217 discriminated CAA patients with and without AD co‐pathology (area under the curve = 0.920) and showed no association with MRI markers of CAA burden. Predefined cut‐offs (≥ 0.27 pg/mL and ≥ 0.34 pg/mL) yielded high accuracy for identifying AD co‐pathology within CAA.

**CONCLUSIONS:**

Plasma p‐tau217 shows high diagnostic accuracy for identifying AD co‐pathology in CAA and is not associated with MRI markers of CAA burden, supporting its specificity for AD‐related pathology.

## Highlights

- Plasma phosphorylated tau (p‐tau)217 measured on an automated clinical platform shows high diagnostic accuracy for detecting Alzheimer's disease (AD) co‐pathology in cerebral amyloid angiopathy (CAA).
- Validation against cerebrospinal fluid amyloid/tau positive biomarkers confirms that plasma p‐tau217 reflects AD‐type tau pathology rather than vascular amyloid burden.
- Plasma p‐tau217 does not associate with magnetic resonance imaging markers of CAA severity, including cerebral microbleeds and cortical superficial siderosis.
- Predefined plasma p‐tau217 cut‐offs (≥ 0.27 pg/mL and particularly ≥ 0.34 pg/mL) accurately discriminate CAA with and without AD co‐pathology.
- Plasma p‐tau217 may support biological stratification of CAA patients according to the presence of AD co‐pathology.

## 1 BACKGROUND

Cerebral amyloid angiopathy (CAA) is a common cause of cognitive impairment and lobar intracerebral hemorrhage in older adults. It is defined by progressive amyloid beta (Aβ) deposition within cortical and leptomeningeal vessels^1^ and is diagnosed in vivo using the Boston v2.0 criteria.^2^ CAA frequently co‐occurs with Alzheimer's disease (AD), characterized by parenchymal Aβ plaques and hyperphosphorylated tau tangles. This overlap generates a biological continuum of mixed vascular and neurodegenerative pathology,^1^, ^3^ which influences clinical presentation, prognosis, and therapeutic vulnerability, including the risk of amyloid‐related imaging abnormalities (ARIAs) during anti‐amyloid therapy.^4^, ^5^ Concomitant AD pathology is associated with greater cognitive burden compared to pure CAA, and recognizing this co‐pathology helps clarify the relative contribution of vascular versus neurodegenerative mechanisms to symptoms. Identifying AD co‐pathology in patients with CAA is therefore clinically relevant, particularly in those presenting with cognitive impairment, in whom overlapping mechanisms may lead to heterogeneous clinical trajectories and complicate both diagnosis and prognostic interpretation.

Cerebrospinal fluid (CSF) biomarkers enable reliable detection of AD‐related amyloid and tau pathology,^6^ but their invasiveness limits use in patients with vascular or hemorrhagic phenotypes. Blood‐based biomarkers have emerged as accessible alternatives. Among them, plasma phosphorylated tau at threonine 217 (p‐tau217) shows high diagnostic accuracy for AD pathology, a strong concordance with CSF biomarkers, particularly CSF p‐tau181 and the CSF Aβ42/p‐tau181 ratio, as well as amyloid positron emission tomography, and has recently been validated on fully automated clinical platforms.^7^, ^8^, ^9^

Although patients with CAA exhibit reduced CSF Aβ40 compared to AD,^10^, ^11^, ^12^, ^13^ no fluid biomarker has been validated for diagnosing CAA itself. Recent studies have reported altered plasma p‐tau isoforms in CAA,^14^, ^15^ but their diagnostic meaning remains unclear. Performance against CSF‐defined AD status using automated assays is lacking. Additionally, the potential correlations between plasma p‐tau217 concentrations and magnetic resonance imaging (MRI) markers of vascular burden–such as cerebral microbleeds (CMBs), cortical superficial siderosis (cSS), and the total CAA–small vessel disease (CAA–SVD) score—remain unexplored.

In this study, we investigated the diagnostic utility of plasma p‐tau217 to detect AD co‐pathology in patients with CAA, using CSF profiles as the biological reference standard. We also examined its associations with CSF core AD biomarkers and vascular MRI markers.

## 2 METHODS

### 2.1 Study design and participants

This study is part of the ANGMAR project (Characterization of Biomarkers Footprint in Cerebral Amyloid Angiopathy Patients),^16^ a prospective observational study embedded within the BIODEGMAR cohort at Hospital del Mar. Participants were recruited from the Neurology Service at Hospital del Mar, including memory‐clinic outpatients (Cognitive and Behavioral Neurology Unit) and Neurovascular Unit. All participants followed the same standardized ANGMAR/BIODEGMAR workflow for clinical assessment, MRI acquisition, and plasma and CSF collection. CAA diagnosis was established on MRI according to Boston criteria v2.0.^2^

For the present analysis, we included individuals with available plasma measurements of p‐tau217, using a fully automated Lumipulse platform (Fujirebio)^9^.

Inclusion criteria were: (1) availability of p‐tau217 plasma measurement; (2) availability of CSF Aβ42, Aβ40, and p‐tau181, quantified using the Lumipulse G600II platform (Fujirebio)^17^; and (3) fulfilment of one of the following diagnostic groups: (i) CAA: probable or possible CAA according to the Boston criteria v2.0^ = ^,^2^ further stratified based on CSF biomarker profiles into CAA with AD co‐pathology (CAA+AD+) or without it (CAA+AD−). AD co‐pathology was defined by a CSF biomarker profile consistent with AD pathology (A+T+)^6^; (ii) patients with mild cognitive impairment (MCI) or AD dementia (AD‐d), fulfilling the clinical criteria established by the National Institute on Aging and the Alzheimer's Association (NIA‐AA, 2011)^18^, ^19^, presenting a CSF biomarker profile consistent with AD pathology (A+T+)^6^ and not meeting criteria for either probable or possible CAA according to the Boston criteria v2.0^2^ (CAA−AD+); and (iii) cognitively unimpaired healthy controls (HCs), characterized by normal neuropsychological performance^20^ and no history of stroke or transient ischemic attack.

All participants underwent standardized clinical, neuropsychological, and MRI evaluations as part of the ANGMAR/BIODEGMAR protocol. Further details regarding diagnostic criteria and biomarker processing are available in the supporting information. The participant flowchart is presented in Figure S1 in supporting information.

### 2.2 Clinical and neuropsychological assessments

All participants underwent a comprehensive clinical and neuropsychological assessment as part of the standardized ANGMAR/BIODEGMAR protocol. Diagnostic classification was based on established international criteria, including the Boston criteria v2.0 for the diagnosis of CAA^2^ and the NIA–AA criteria for MCI^18^ and AD‐d^19^.

Cognitive and functional status were evaluated using the Mini‐Mental State Examination (MMSE)^21^ and the Clinical Dementia Rating (CDR) scale total score^22^. Detailed procedures are described in the Supplementary Methods in supporting information.

**RESEARCH IN CONTEXT**

1. **Systematic review**: We reviewed the literature on plasma phosphorylated tau (p‐tau) biomarkers in Alzheimer's disease (AD) and cerebral amyloid angiopathy (CAA). Plasma p‐tau217 has shown high diagnostic accuracy for AD and strong concordance with cerebrospinal fluid (CSF) biomarkers and amyloid positron emission tomography. However, evidence regarding its performance in patients with CAA, particularly using automated clinical platforms and CSF‐defined reference standards, remains limited.
2. **Interpretation**: In this study, plasma p‐tau217 measured on an automated clinical platform accurately detected AD co‐pathology in patients with CAA when validated against CSF amyloid/tau positive biomarkers. Plasma p‐tau217 showed no association with magnetic resonance imaging markers of vascular amyloid burden, indicating specificity for AD‐type tau pathology rather than CAA‐related vascular injury.
3. **Future directions**: Plasma p‐tau217 may serve as a non‐invasive tool to biologically stratify patients with suspected CAA and mixed vascular–neurodegenerative phenotypes. Future longitudinal and multicenter studies should evaluate its prognostic value and explore complementary biomarkers targeting vascular amyloid pathology to improve comprehensive biological characterization.

### 2.3 Plasma and CSF biomarkers analysis

Plasma p‐tau217 concentrations were measured using the Lumipulse pTau217 Plasma assay on fully automated Lumipulse analyzers (G600II or G1200; Fujirebio), as previously described^9^, ^23^. Measurements were performed at two certified laboratories—the Barcelonaβeta Brain Research Center (BBRC) and the Laboratori de Referència de Catalunya (LRC)—following standardized laboratory procedures. Plasma samples were processed and stored according to standardized protocols, including centrifugation, aliquoting, and storage at −80°C until analysis.

Plasma p‐tau217 values were classified into three‐level groups according to cutoff points previously validated in the multicenter study by Palmqvist et al.:^9^ high (> 0.34 pg/mL, consistent with AD), intermediate (0.22–0.34 pg/mL), and low (< 0.22 pg/mL). For the main diagnostic accuracy analysis against the CSF reference standard, a single cutoff of > 0.27 pg/mL was applied to classify plasma p‐tau217 values as compatible with AD.

CSF biomarkers were determined at the LRC using the fully automated Lumipulse G600II analyzer (Fujirebio)^17^. The analytical panel included Aβ42, Aβ40, p‐tau181, and total tau (t‐tau). Cutoff values for individual biomarkers and derived ratios were based on previously validated thresholds^24^: Aβ42, 750 pg/ml; Aβ42/Aβ40 ratio, 0.062; p‐tau181, 69.85 pg/ml; t‐tau, 522.0 pg/ml. AD biological status (A+T+) was defined using the Aβ42/Aβ40 ratio and p‐tau181 according to these predefined thresholds, and was used as the reference standard to evaluate the diagnostic performance of plasma p‐tau217. All included participants had paired plasma and CSF samples collected on the same day.

### 2.4 Neuroimaging

Structural brain MRI was acquired using a 3T scanner (Philips Achieva). The imaging protocol included T1‐ and T2‐weighted sequences, high‐resolution 3D T1, diffusion‐weighted imaging (DWI), fluid‐attenuated inversion recovery (FLAIR), and susceptibility‐sensitive sequences including both T2*‐weighted gradient echo (T2*‐GRE) and susceptibility‐weighted imaging (SWI).

All MRI scans underwent standardized clinical neuroradiological evaluation and were subsequently reviewed by an experienced neuroradiologist participating in the study, blinded to clinical and biomarker data. CAA‐related pathology was assessed in all participants based on the presence and severity of lobar CMBs and cSS, evaluated on T2*‐GRE; convexity subarachnoid hemorrhage (cSAH), evaluated on FLAIR and T2*‐GRE; white matter hyperintensities (WMHs), assessed on FLAIR and considered significant when the Fazekas score^25^ was ≥ 2; and enlarged perivascular spaces in the centrum semiovale (CSO‐PVS), rated on T2‐weighted sequences. For CAA classification according to the Boston criteria v2.0,^2^the multispot WMH pattern (defined as > 10 small circular or ovoid hyperintense lesions in the subcortical white matter of both hemispheres) was assessed as a non‐hemorrhagic marker independently from Fazekas scoring. CMB burden was considered significant when there were ≥ 5 lobar CMBs, according to previous CAA neuroimaging studies^26^, ^27^. The CAA–SVD burden score was calculated to quantify the overall degree of CAA‐related small‐vessel disease.

### 2.5 Ethics

The study protocols for both the ANGMAR project and the BIODEGMAR cohort were approved by the ethics committee of the Hospital del Mar Research Institute (project codes 2021/9960/I and 2018/7805/I, respectively). All participants, or their legal representatives when applicable, provided written informed consent prior to inclusion. The study was conducted in accordance with the principles of the Declaration of Helsinki.

### 2.6 Statistical analyses

#### 2.6.1 Descriptive analyses and clinical correlates of p‐tau217

Variables were summarized as means (standard deviations), medians (interquartile ranges [IQRs]), or frequencies (percentages), depending on their distribution. Differences in clinical characteristics between diagnostic groups were assessed using *t* tests, Kruskal–Wallis tests, or chi‐square tests, as appropriate. We then examined the distribution of plasma p‐tau217 across clinical diagnoses and evaluated its correlation with AD core biomarkers. We also assessed whether plasma p‐tau217 was associated with other relevant clinical variables, including apolipoprotein E (*APOE*) ε2 carriers, *APOE* ε4 carriers, and vascular risk factors.

In our cohort, p‐tau217 levels displayed a skewed distribution. Therefore, for univariate analyses we used non‐parametric tests (Kruskal–Wallis and Spearman rank correlation test). For multivariate analyses that might be affected by influential cases we used the log‐transform variable (Figure S2 in supporting information).

#### 2.6.2 Differences in plasma p‐tau217 across groups

To assess whether plasma p‐tau217 levels differed between groups independently of potential confounders, we constructed an analysis of covariance (ANCOVA) model with log p‐tau217 as the dependent variable and clinical diagnosis as the grouping factor, adjusting for age and sex. Marginal means and standard errors were then estimated, and pairwise comparisons were performed with Bonferroni correction. Effect sizes were reported using Cohen |*d*|. We additionally conducted sensitivity analyses excluding participants with possible CAA to assess the robustness of our results.

#### 2.6.3 Diagnostic performance of plasma p‐tau217

As a next step, we evaluated the diagnostic performance of plasma p‐tau217 for identifying AD in a cohort enriched with patients with CAA, with or without concomitant AD. We categorized p‐tau217 using three cutoff values as described above (see section 2.3):^9^ (1) > 0.22 pg/mL, (2) > 0.27 pg/mL, and (3) > 0.34 pg/mL. We then compared the area under the receiver operating characteristic curve (AUC) of continuous p‐tau217 and these categorical cutoffs across the following contrasts: (1) AD+ versus AD−; (2) CAA+ versus CAA−; (3) CAA+AD− versus CAA−AD+; and (4) CAA+AD+ versus CAA+AD−.

To further identify the best‐performing cutoff, we performed bootstrap resampling (*K* = 2000) with replacement from the original cohort. For each resampled dataset, we calculated accuracy, AUC, recall, precision, and specificity, generating empirical distributions of these metrics. From these, we derived 95% confidence intervals.

Using this bootstrap framework, we also quantified the pairwise differences in these metrics across cutoffs: > 0.22 pg/mL versus > 0.27 pg/mL; > 0.22 pg/mL versus > 0.34 pg/mL; and > 0.27 pg/mL versus > 0.34 pg/mL. For each resampling, we computed the Δ for each metric, enabling statistical inference on whether specific cutoffs yielded significant improvements in performance using the same set of metrics.

#### 2.6.4 Associations between plasma p‐tau217 and MRI markers

Associations between plasma p‐tau217 concentrations and MRI markers of CAA burden—including the number of CMBs, presence and extent of cSS, WMHs, and the overall CAA–SVD burden score—were examined to explore the relationship between plasma tau levels and vascular amyloid load. These analyses were restricted to participants fulfilling Boston v2.0 criteria for probable or possible CAA (with or without AD co‐pathology). To this end, we constructed ANCOVA models with plasma log p‐tau217 as the dependent variable and each MRI marker of CAA entered as the predictor of interest in separate models. All models were adjusted for age and sex.

## 3 RESULTS

### 3.1 Demographic and baseline characteristics

A total of 231 participants were studied, including 27 cognitively unimpaired HCs, 50 patients with CAA (21 CAA+AD− and 29 CAA+AD+), and 154 participants with AD without CAA (CAA−AD+; Figure S1). Age and sex distributions did not differ significantly between groups (age, *p* = 0.082; sex, *p* = 0.062), whereas education attainment was highest in HCs (*p* = 0.013). The proportion of *APOE* ε4 carriers differed significantly among groups (*p* < 0.001), with higher frequencies in CAA+AD+ (74.1%) and CAA−AD+ (60.7%) than in HCs (20.8%) and CAA+AD− (15.8%). Group comparisons for demographic and clinical variables are detailed in Table 1. Additional comparisons of the clinical and radiological features of the CAA subgroups are presented in Table S1 in supporting information, while Table S2 in supporting information compares participant characteristics between the possible and probable CAA diagnostic groups.

**TABLE 1:** Participants’ characteristics.

	All (*n* = 231)	HC (*n* = 27)	CAA+AD– (*n* = 21)	CAA+AD+ (*n* = 29)	CAA–AD+ (*n* = 154)	*p* value
Age, years	73.0 [69.0;76.0]	72.0 [62.5;74.5]	72.0 [66.0;75.0]	75.0 [71.0;77.0]	73.0 [70.0;76.0]	0.082
Sex, *n* (%)						0.062
Female	137 (59.3%)	13 (48.1%)	12 (57.1%)	12 (41.4%)	100 (64.9%)	
Male	94 (40.7%)	14 (51.9%)	9 (42.9%)	17 (58.6%)	54 (35.1%)	
Ethnicity, *n* (%)						0.154
White	224 (97.4%)	26 (100%)	19 (90.5%)	28 (96.6%)	151 (98.1%)	
Other	6 (2.61%)	0 (0.00%)	2 (9.52%)	1 (3.45%)	3 (1.95%)	
Education, years	8.00 [6.00; 10.0]	8.00 [8.00; 14.0]	8.00 [8.00; 12.0]	8.00 [6.00; 10.5]	8.00 [6.00; 8.75]	0.013
Hypertension, *n* (%)	129 (55.8%)	9 (33.3%)	12 (57.1%)	19 (65.5%)	89 (57.8%)	0.075
Diabetes mellitus, *n* (%)	45 (19.5%)	3 (11.1%)	2 (9.52%)	7 (24.1%)	33 (21.4%)	0.387
Dyslipidemia, *n* (%)	129 (56.1%)	12 (44.4%)	8 (38.1%)	18 (64.3%)	91 (59.1%)	0.134
Obesity, *n* (%)	25 (14.6%)	2 (15.4%)	5 (26.3%)	2 (8.33%)	16 (13.9%)	0.428
Ischemic heart disease, *n* (%)	12 (5.19%)	2 (7.41%)	0 (0.00%)	2 (6.90%)	8 (5.19%)	0.691
Antiplatelet therapy, *n* (%)	50 (21.6%)	4 (14.8%)	3 (14.3%)	9 (31.0%)	34 (22.1%)	0.438
MMSE	22.0 [18.0; 25.0]	29.0 [27.2; 30.0]	25.5 [22.5; 28.0]	20.5 [17.2; 24.0]	21.0 [18.0; 23.0]	< 0.001
CDR						
0	35 (15.2%)	27 (100%)	7 (33.3%)	1 (3.45%)	0 (0.00%)	
0.5	62 (26.8%)	0 (0.00%)	7 (33.3%)	9 (31.0%)	46 (29.9%)	
1	63 (27.3%)	0 (0.00%)	1 (4.76%)	1 (3.45%)	61 (39.6%)	
2	59 (25.5%)	0 (0.00%)	4 (19.0%)	15 (51.7%)	40 (26.0%)	
3	12 (5.19%)	0 (0.00%)	2 (9.52%)	3 (10.3%)	7 (4.55%)	
Fazekas score						
0 (*n*, %)	46 (20.1%)	10 (40.0%)	2 (9.52%)	5 (17.2%)	29 (18.8%)	
1 (*n*, %)	109 (47.6%)	13 (52.0%)	6 (28.6%)	6 (20.7%)	84 (54.5%)	
≥2 (*n*, %)	74 (32.3%)	2 (8.00%)	13 (61.9%)	18 (62.1%)	41 (26.6%)	
*APOE* ε4 carriers, *n* (%)	99 (52.9%)	5 (20.8%)	3 (15.8%)	20 (74.1%)	71 (60.7%)	< 0.001
*APOE* ε2 carriers, *n* (%)	18 (9.63%)	2 (8.33%)	5 (26.3%)	3 (11.1%)	8 (6.84%)	0.072

*Note*: Data are expressed as median and interquartile range or percentage (%). Education was unavailable in 1 participant, obesity in 60 participants, MMSE in 5 participants, Fazekas score in 2 participants, and *APOE* genotype in 44 participants.

Abbreviations: AD, Alzheimer's disease; *APOE*, apolipoprotein E; CAA, cerebral amyloid angiopathy; CDR, Clinical Dementia Rating; HC, healthy control; MMSE, Mini‐Mental State Examination.

### 3.2 Clinical correlates of plasma p‐tau217

When examining the associations between demographic and clinical variables and plasma p‐tau217, we observed a weak inverse correlation with years of education (*ρ* = −0.15, *p* = 0.025). Higher plasma p‐tau217 was also associated with dyslipidemia and *APOE* ε4 carrier status (Figure S3 and Table S3 in supporting information).

**FIGURE 1:**
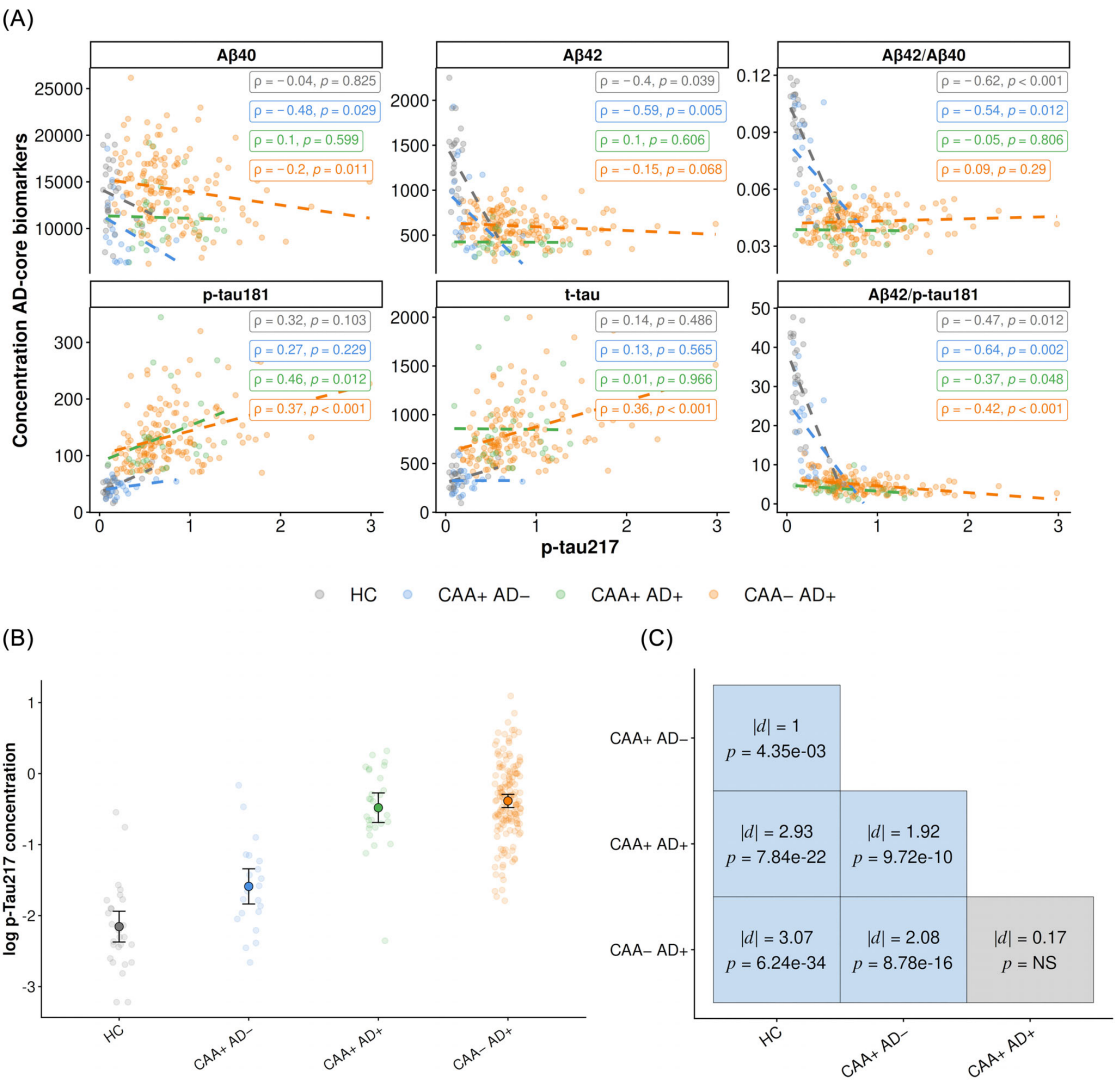
CSF AD‐core biomarker correlates of plasma p‐tau217 and group differences. A, Correlations between CSF AD core biomarkers and plasma p‐tau217 levels. Colors represent diagnostic groups. Correlation coefficients were obtained using Spearman rank correlation test. B, Marginal means from an ANCOVA model examining differences in log‐transformed plasma p‐tau217 across diagnostic groups, adjusted for age and sex. C, Pairwise comparisons derived from the ANCOVA model. Blue tiles indicate comparisons that remained significant after Bonferroni correction. For each comparison, Cohen |*d*| and corresponding *p* values are shown. Aβ, amyloid beta; AD, Alzheimer's disease; ANCOVA, analysis of covariance; CAA, cerebral amyloid angiopathy; CSF, cerebrospinal fluid; HC, healthy control; p‐tau, phosphorylated tau; t‐tau, total tau

We next assessed the associations between CSF AD core biomarkers and plasma p‐tau217. Plasma p‐tau217 showed positive correlations with CSF p‐tau181 in participants with CAA**−**AD+ (*ρ* = 0.37, *p* < 0.001) and CAA+AD+ (*ρ* = 0.46, *p* = 0.012; Figure 1A). Plasma p‐tau217 correlated with CSF t‐tau in participants with CAA**−**AD+ (*ρ* = 0.36, *p* < 0.001). Across diagnostic groups, plasma p‐tau217 was consistently inversely associated with the CSF Aβ42/p‐tau181 ratio (*p* < 0.05 in all cases), consistent with greater AD‐related pathology. In contrast, associations between plasma p‐tau217 and CSF amyloid measures alone (Aβ40, Aβ42, and Aβ42/Aβ40) were heterogeneous and did not show a consistent pattern across diagnostic groups.

### 3.3 Characterization of plasma p‐tau217 across diagnostic groups

Mean plasma p‐tau217 differed significantly across diagnostic groups, with lowest levels among HCs, intermediate in individuals with CAA+AD−, and highest in participants with CAA+AD+ and CAA**−**AD+. After adjusting for age and sex, we detected significant overall differences among groups (*p* < 0.001; Figure 1B). Post hoc pairwise tests showed no significant differences between CAA**−**AD+ and CAA+AD+ participants. In contrast, modest but significant differences were observed between HC and CAA+AD**−** groups (Cohen *|d*| = 1.00, *p* < 0.05; Figure 1C). All other group comparisons demonstrated larger effect sizes, with elevated plasma p‐tau217 in CAA+AD+ and CAA**−**AD+ relative to HC and CAA+AD**−** (Cohen |*d*| ranging from 1.92 to 3.07, *p* < 0.001; Figure 1C). Plasma p‐tau217 distributions by diagnostic group are shown in Figure S4 and Table S4 in supporting information.

We further assessed whether these findings were influenced by the classification of CAA as possible or probable. Table S5 in supporting information compares p‐tau217 levels across patients with possible and probable CAA, stratified according to the presence or absence of AD co‐pathology. Importantly, p‐tau217 levels did not differ significantly between patients with possible and probable CAA, indicating that the observed associations were not driven by CAA diagnostic category (Figure S5 in supporting information). In addition, we performed sensitivity analyses of the multivariable models after excluding participants with possible CAA and obtained equivalent results as those reported in the whole sample (Figure S6 in supporting information).

### 3.4 Diagnostic performance of plasma p‐tau217

**FIGURE 2:**
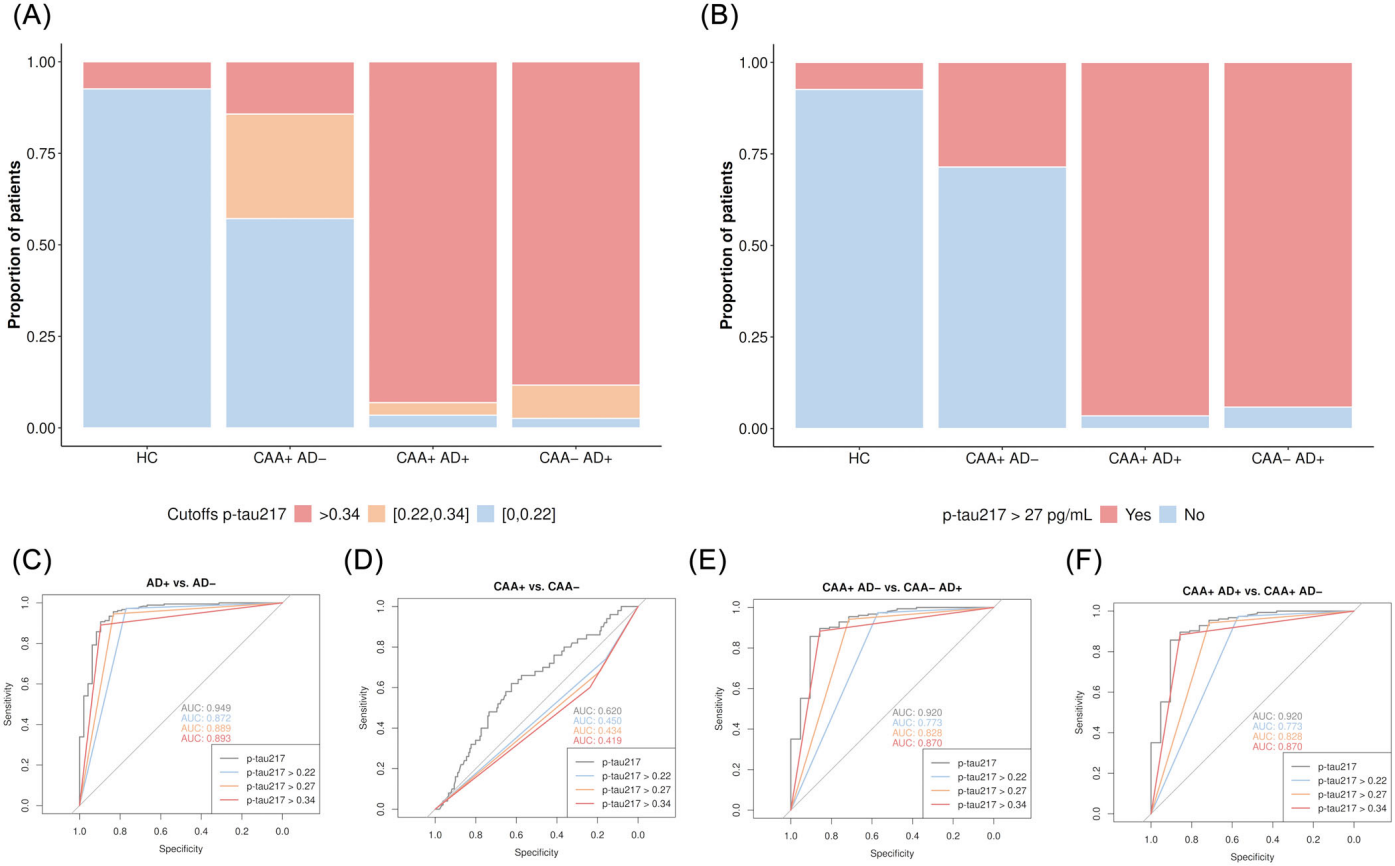
Performance of p‐tau217 for detecting AD in a cohort enriched with CAA participants. A, Proportion of participants with elevated plasma p‐tau217 according to predefined cutoffs (> 0.22 and > 0.34 pg/mL). B, Proportion of participants with elevated p‐tau217 according to the > 0.27 pg/mL cutoff. C–F, Receiver operating curves for continuous and categorized p‐tau217 across four clinically relevant diagnostic contrasts: (C) AD+ versus AD− (entire cohort), (D) CAA+ versus CAA−, (E) CAA+AD− versus CAA−AD+, (F) CAA+AD+ versus CAA+AD−. AUC values for continuous and categorized p‐tau217 (> 0.22, > 0.27, and > 0.34 pg/mL) are displayed within each panel. AD, Alzheimer's disease; AUC, area under the receiver operating characteristic curve; CAA, cerebral amyloid angiopathy; HC, healthy control; p‐tau, phosphorylated tau

When categorizing plasma p‐tau217 according to predefined clinical cutoffs, 93.1% of CAA+AD+ and 88.3% of CAA**−**AD+ participants were classified as high (> 0.34 pg/mL), whereas only 7.4% of HC and 14.2% of CAA+AD− exceeded this threshold. Intermediate values (0.22–0.34 pg/mL) were observed in 0% of HC, 28.6% of CAA+AD**−**, 3.4% of CAA+AD+, and 9.1% of CAA**−**AD+ (Figure 2A) participants. Using a single cutoff of 0.27 pg/mL, 96.5% of CAA+AD+ and 94.1% of CAA**−**AD+ participants were above this threshold, whereas 7.4% of HC and 28.5% of CAA+AD**−** exceeded it (Figure 2B).

Diagnostic performance was evaluated for four clinically relevant contrasts: AD+ versus AD−, CAA+ versus CAA**−**, CAA+AD**−** versus CAA**−**AD+, and CAA+AD+ versus CAA+AD**−**. Continuous plasma p‐tau217 demonstrated excellent discrimination of AD across contrasts, with an AUC of 0.949 for AD+ versus AD**−**, 0.920 for CAA+AD**−** versus CAA**−**AD+, and 0.920 for CAA+AD+ versus CAA+AD**−** (Figures 2C–2F). In contrast, discrimination of CAA irrespective of AD was poor (AUC = 0.620; Figure 2D). Categorizing p‐tau217 using predefined cutoffs (> 0.22, > 0.27, and > 0.34 pg/mL) maintained high diagnostic accuracy for AD‐related contrasts (AUC range 0.872–0.893 for AD+ vs. AD**−**; Figure 2C). In contrasts involving CAA patients (CAA+AD**−** vs. CAA**−**AD+ and CAA+AD+ vs. CAA+AD**−**), the > 0.22 pg/mL cutoff yielded lower discrimination (AUC 0.773 for both comparisons), whereas performance improved with the > 0.27 and > 0.34 pg/mL thresholds (Figures 2E and 2F).

**FIGURE 3:**
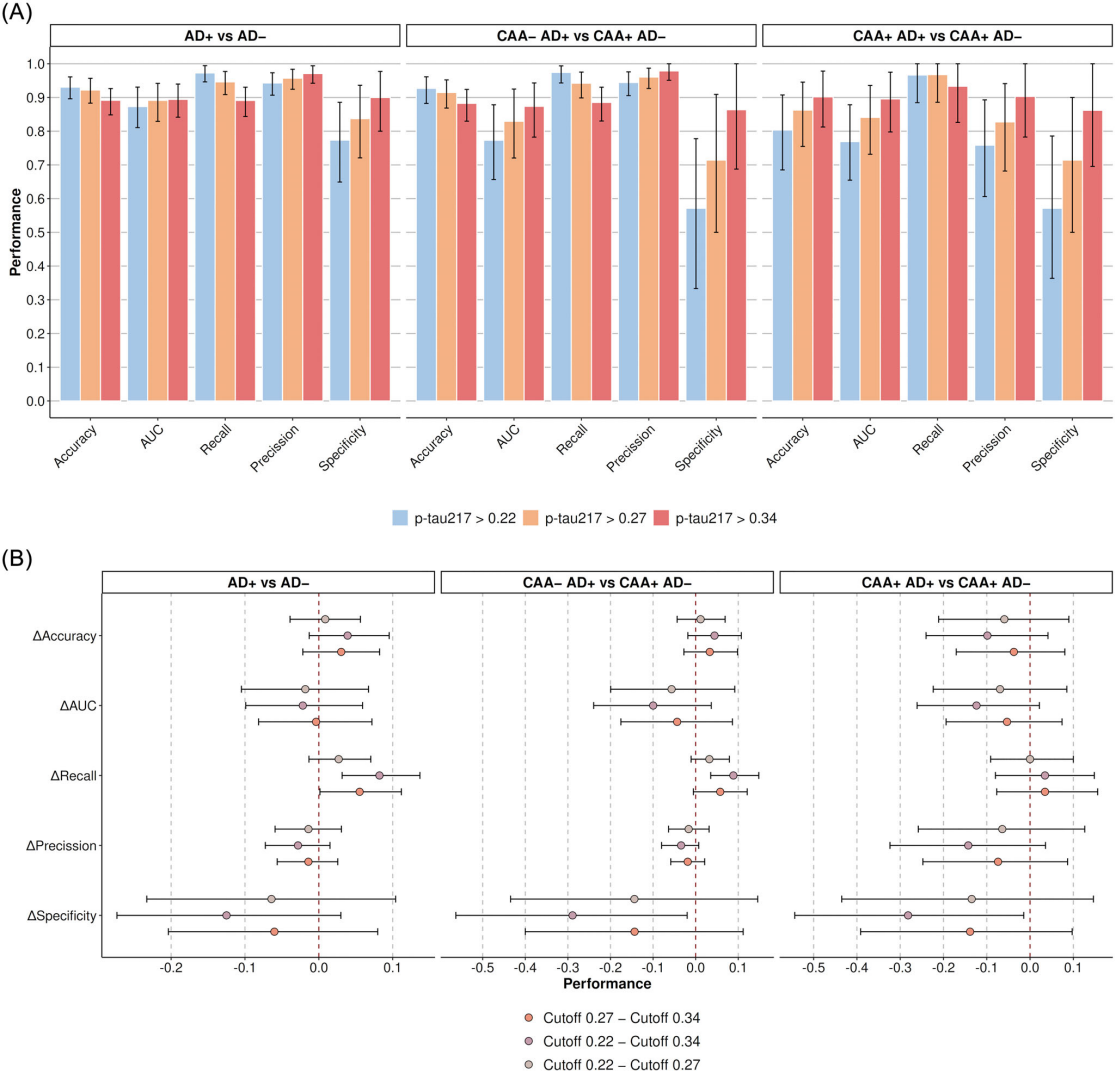
Bootstrap analysis comparing p‐tau217 cutoffs for detecting AD across groups. A, Bootstrap‐derived performance of categorized p‐tau217 for detecting AD in different settings: the entire cohort, participants without co‐pathology, and those with CAA. The *y* axis represents the average bootstrapped value for each performance metric (shown on the *x* axis). Error bars indicate 95% confidence intervals. B, Pairwise comparisons of p‐tau217 cutoffs, based on the differences in each metric computed from every resampled dataset. Colors represent each contrast (> 0.22 vs. > 0.27, > 0.22 vs. > 0.34, and > 0.27 vs. > 0.34), and panels correspond to the same settings as in (A). Dots indicate the mean Δ for each metric, and error bars represent 95% confidence intervals. Precision corresponds to the positive predictive value (PPV), recall to sensitivity, and accuracy to the proportion of correctly classified cases. AD, Alzheimer's disease; AUC, area under the receiver operating characteristic curve; CAA, cerebral amyloid angiopathy; p‐tau, phosphorylated tau

We then performed a bootstrap analysis to evaluate the robustness of plasma p‐tau217 performance across all cutoffs for detecting AD‐related pathology. In the overall cohort (AD+ vs. AD**−**), AUC values remained consistently high across thresholds, with the > 0.34 pg/mL cutoff increasing specificity compared to > 0.22 pg/mL, at the cost of a modest reduction in recall (Figure 3). The > 0.27 pg/mL cutoff provided the most balanced trade‐off between recall and specificity. Comparing patients with CAA+AD− versus CAA−AD+, the > 0.34 pg/mL cutoff was associated with higher specificity relative to > 0.22 pg/mL, with only a limited reduction in recall (Figure 3). In this comparison, differences between the > 0.27 and > 0.34 pg/mL thresholds were not statistically significant in the bootstrap analysis. Finally, in the most challenging contrast (CAA+AD+ vs. CAA+AD−), the > 0.34 pg/mL cutoff preserved higher specificity without a statistically significant decrease in recall compared to > 0.22 pg/mL (Figure 3).

### 3.5 Associations between plasma p‐tau217 and MRI markers

We subsequently explored the relationship between plasma p‐tau217 concentrations and MRI features indicative of vascular amyloid burden. These analyses were restricted to participants fulfilling Boston v2.0 criteria for probable or possible CAA, regardless of the presence of AD co‐pathology. Table S1 summarizes the radiological characteristics of the CAA+AD− and CAA+AD+ groups. No significant group differences were observed for any MRI markers, including the number of lobar CMBs (*p* = 0.21), presence of cSS (*p* = 0.25), WMHs (*p* = 0.71), or the global CAA–SVD burden score (*p* = 0.92).

Within the overall CAA subgroup, we next assessed the relationship between plasma p‐tau217 concentrations and CAA‐related MRI markers. In ANCOVA models adjusted for age and sex, plasma p‐tau217 showed no significant associations with either the CAA–SVD burden score or the individual MRI markers of CAA severity (all P values* > *0.05; Figures S7 and S8 in supporting information).

## 4 DISCUSSION

This study demonstrates high diagnostic accuracy of plasma p‐tau217 for identifying AD pathology irrespective of concomitant CAA, while only modestly increased concentrations are observed in CAA without AD. Plasma p‐tau217 discriminated AD+ from AD− participants with high accuracy but performed poorly for detecting CAA itself. Higher cutoffs improved specificity within CAA populations, and no association was observed between plasma p‐tau217 and MRI markers of CAA severity.

To our knowledge, this is the first study to assess the diagnostic performance of plasma p‑tau217 on a fully automated clinical platform against CSF‑defined AD status within an MRI‑defined CAA cohort. Using CSF biomarkers as the biological reference standard, plasma p‐tau217 accurately discriminated cases with AD co‐pathology (CAA+AD+) from those with pure CAA (CAA+AD−). These findings support that plasma p‐tau217 primarily reflects AD‐related amyloid and tau pathology rather than vascular amyloid deposition, consistent with previous observations of intermediate plasma p‐tau217 concentrations in CAA^15^.

Plasma p‐tau217 correlated with CSF markers of AD pathology. Associations with CSF p‐tau181 were observed in individuals with AD pathology irrespective of CAA status, and plasma p‐tau217 was consistently inversely associated with the CSF Aβ42/p‐tau181 ratio. In contrast, correlations with CSF amyloid measures alone were heterogeneous across diagnostic groups. Together, these findings support that plasma p‐tau217 reflects AD‐specific pathophysiological processes and remains independent of CAA‐related vascular burden.

Plasma p‐tau217 concentrations were significantly elevated in individuals with AD regardless of the presence of concomitant CAA, whereas pure CAA cases showed only slight increases compared to HC. Consistently, no differences were observed between CAA+AD+ and CAA−AD+, reinforcing that p‐tau217 reflects AD‐type molecular changes even in the presence of CAA. Notably, 28.6% of CAA+AD− participants exhibited intermediate p‐tau217 values. These may reflect subtle tau phosphorylation related to vascular dysfunction or impaired protein clearance^1^, ^3^, as suggested in recent CAA studies^15^, although very early AD‐type changes not yet detectable by CSF p‐tau181 cannot be excluded, given the higher analytical sensitivity of plasma p‐tau217 to early tau phosphorylation.^7^, ^8^ Recent evidence suggests that plasma p‐tau217 is increasingly viewed as a marker of amyloid‐associated tau phosphorylation rather than an exclusively AD‐specific biomarker^7^. Accordingly, intermediate plasma p‐tau217 values observed in a subset of CAA+AD− individuals may reflect low‐level amyloid‐related tau phosphorylation within the vascular amyloid spectrum, although concentrations remained substantially lower than those observed in biologically defined AD.

From a clinical perspective, bootstrap analyses indicated that the 0.27 pg/mL cutoff provided the best balance between sensitivity and specificity in unselected cohorts, whereas in patients with suspected CAA, using the higher 0.34 pg/mL threshold may provide a more conservative and specific rule‐in strategy for detecting AD co‐pathology. The absence of any association between plasma p‐tau217 and MRI markers of CAA severity underscores its independence from vascular amyloid burden and is consistent with previous studies showing no association between plasma p‐tau isoforms and CMBs^28^.

Taken together, these findings indicate that plasma p‐tau217 is a marker of AD co‐pathology within the CAA continuum, rather than a biomarker of CAA itself. Our results extend previous CSF‐based evidence and provide the first systematic evaluation of plasma p‐tau217 in a CSF‐defined CAA cohort, supporting its translational utility for identifying AD co‐pathology in patients with CAA.

### 4.1 Clinical implications

The clinical relevance of these findings lies in the increasing need to distinguish CAA without AD co‐pathology (CAA+AD−) from CAA with concomitant AD pathology (CAA+AD+), conditions that often overlap clinically but diverge biologically. In patients with cognitive impairment and a substantial vascular burden, determining the relative contribution of vascular and AD‐related mechanisms remains challenging. Plasma p‐tau217 offers a non‐invasive tool to identify underlying AD pathology in this setting, complementing MRI‐based diagnostic criteria and facilitating biological characterization.

Identifying AD co‐pathology in patients with CAA has important prognostic implications, as concomitant AD pathology is associated with earlier onset and faster progression of cognitive decline^6^. Although patients with CAA are currently considered at high risk for ARIAs and are often excluded from anti‐amyloid monoclonal antibody therapy^29^, ^30^, early detection of AD pathology remains clinically meaningful, helping refine expectations regarding disease trajectory, guide individualized care planning, and inform shared decision making. The absence of associations between plasma p‐tau217 and MRI markers of CAA severity indicates that this biomarker should not be used to estimate hemorrhagic risk, but rather to identify concomitant AD‐related pathology.

Beyond clinical care, plasma p‐tau217 may enhance research stratification by distinguishing mixed CAA+AD+ from isolated vascular forms. The observation of intermediate p‐tau217 values in a subset of CAA+AD− individuals highlights the need for longitudinal follow‐up to determine whether these patterns reflect subthreshold AD‐related tau phosphorylation or variability within the vascular amyloid spectrum. These findings further underscore the need for dedicated vascular‐specific biomarkers capable of capturing CAA‐related biological processes.

### 4.2 Strengths

This study has several notable strengths. First, it provides the first evaluation of plasma p‐tau217 in a CAA cohort with a CSF‐defined AD biomarker profile, ensuring a biologically grounded classification of AD co‐pathology. Second, plasma and CSF biomarkers were quantified using a fully automated Lumipulse platform, minimizing pre‐analytical variability and enabling reliable cross‐fluid comparisons. Third, the cohort spans the clinical and biological spectrum of CAA, alongside biologically defined AD cases and cognitively unimpaired controls, allowing characterization of plasma p‐tau217 across a broad spectrum of vascular and neurodegenerative pathology.

### 4.3 Limitations

This study is not free of limitations. First, its cross‐sectional design precludes conclusions about the temporal dynamics or prognostic value of plasma p‐tau217 in CAA. Longitudinal follow‐up will be necessary to determine whether elevated or intermediate plasma levels predict cognitive decline or transition from CAA without AD co‐pathology (CAA+AD−) to CAA with concomitant AD pathology (CAA+AD+).

Second, although the overall cohort size is comparable to contemporary single‐center CAA studies, subgroup stratification—particularly within the CAA+AD− group—resulted in modest sample sizes that may limit statistical power and generalizability.

Third, the absence of neuropathological confirmation remains a constraint. While CSF biomarkers provided a robust biological reference standard for AD pathology, CAA diagnosis relied on the clinicoradiological Boston v2.0 criteria. *Post mortem* studies would be required to confirm cerebrovascular amyloid deposition and strengthen clinicopathological correlations.

Finally, plasma p‐tau217 measurements were performed in two different certified laboratories. Although identical analytical technology, standardized reagents, and harmonized preanalytical procedures were used across sites, some degree of inter‐laboratory variability cannot be completely excluded.

## 5 CONCLUSION

In summary, plasma p‐tau217 measured on a fully automated clinical platform shows high diagnostic accuracy for identifying AD co‐pathology within the CAA spectrum. Elevated p‐tau217 concentrations specifically reflect AD‐related tau phosphorylation, whereas pure CAA cases exhibit low or intermediate values that remain clearly below the range observed in CAA+AD+ and CAA−AD+. Plasma p‐tau217 did not distinguish CAA+AD+ from CAA−AD+ and showed no association with vascular amyloid burden or MRI markers of CAA severity, supporting that it reflects concomitant AD pathology rather than CAA itself.

Applying predefined cut‐offs—particularly ≥ 0.34 pg/mL—improved diagnostic precision and provides practical thresholds for clinical interpretation in suspected CAA cases. Taken together, these findings support plasma p‐tau217 as a non‐invasive and biologically specific biomarker for detecting AD co‐pathology in patients with CAA and improving the stratification of mixed vascular–amyloid phenotypes. Replication in larger, multisite cohorts will be essential to confirm generalizability and support broader clinical implementation.

## CONFLICT OF INTEREST STATEMENT

Marc Suárez‐Calvet has received in the past 36 months consultancy/speaker fees (paid to the institution) from by Almirall, Eli Lilly, Quanterix, Novo Nordisk, and Roche Diagnostics. He has received consultancy fees or served on advisory boards (paid to the institution) of Eli Lilly, Grifols, Novo Nordisk, and Roche Diagnostics. He was granted a project and is a site investigator of a clinical trial (funded to the institution) by Roche Diagnostics. In‐kind support for research (to the institution) was received from ADx Neurosciences, Alamar Biosciences, ALZpath, Avid Radiopharmaceuticals, Eli Lilly, Fujirebio, Janssen Research & Development, Meso Scale Discovery, and Roche Diagnostics; M.S.‐C. did not receive any personal compensation from these organizations or any other for‐profit organization. Albert Puig‐Pijoan has served on advisory boards for Schwabe Farma Iberica. All other authors declare no conflicts of interest related to this manuscript. Author disclosures are available in the Supporting Information.

## CONSENT STATEMENT

The ANGMAR study and the BIODEGMAR cohort were approved by the local ethics committee (CEIm Hospital del Mar Research Institute, project codes 2021/9960/I and 2018/7805I, respectively). All participants signed a specific informed consent form.

## Supporting information



Supporting information: alz71709‐supp‐0001‐SuppMat.docx

Supporting information: alz71709‐supp‐0002‐Disclosureforms.pdf
