# Supplementary material for: Plasma p‐tau217 detects Alzheimer's disease co‐pathology in cerebral amyloid angiopathy: Comparison to CSF biomarkers in the ANGMAR cohort
Source: Alzheimers Dement. 2026 Aug 4;22(8):e71709. doi: 10.1002/alz.71709 (PMC13438190; doi:10.1002/alz.71709)
Supplement: Supplementary file 2 [file ALZ-22-e71709-s001.docx]

**Supplementary material**

Fernández-Lebrero A, Jiménez-Balado J *et al*. Plasma p-tau217 detects Alzheimer’s disease co-pathology in cerebral amyloid angiopathy: Comparison with CSF biomarkers in the ANGMAR cohort

**Table of Contents:**

[Supplementary Methods	3](#_Toc231752969)

[Study participants, the DEGMAR register, and the BIODEGMAR study	3](#_Toc231752970)

[BIODEGMAR protocol and procedures	4](#_Toc231752971)

[CSF and plasma collection and storage	6](#_Toc231752972)

[AD CSF core biomarker measurements and cutoff values	6](#_Toc231752973)

[Plasma p-tau217 measurements and cutoff values	7](#_Toc231752974)

[Protocol approvals, registrations, and participant consent	9](#_Toc231752975)

[Supplementary Results	10](#_Toc231752976)

[Clinical and radiological profile of CAA subgroups	10](#_Toc231752977)

[Plasma p-tau217 association with demographic, clinical, and genetic variables	10](#_Toc231752978)

[Supplementary Figures	11](#_Toc231752979)

[Figure S1. Study flowchart	11](#_Toc231752980)

[Figure S2. Distribution of p-tau217 in the ANGMAR cohort	12](#_Toc231752981)

[Figure S3. Clinical correlates of plasma p-tau217	12](#_Toc231752982)

[Figure S4. Distribution of log p-tau217 across diagnostic groups	13](#_Toc231752983)

[Figure S5. Differences in plasma p-tau217 by possible and probable CAA	14](#_Toc231752984)

[Figure S6. Differences in plasma p-tau217 by groups excluding possible CAA	15](#_Toc231752985)

[Figure S7. Differences in plasma p-tau217 by accumulation of MRI CAA markers	16](#_Toc231752986)

[Figure S8. Differences in plasma p-tau217 by individual MRI CAA markers	17](#_Toc231752987)

[Supplementary Tables	18](#_Toc231752988)

[Table S1. CAA participants’ clinical and radiological characteristics	18](#_Toc231752989)

[Table S2. Main characteristics of patients with CAA stratified by possible and probable diagnoses and presence of AD	19](#_Toc231752990)

[Table S3. Univariate associations between plasma p-tau217 concentrations and demographic and clinical variables	20](#_Toc231752991)

[Table S4. Descriptive statistics of plasma and CSF biomarkers by diagnostic group	21](#_Toc231752992)

[Table S5. Descriptive statistics of plasma and CSF biomarkers of patients with CAA stratified by possible and probable diagnoses and presence of AD	21](#_Toc231752993)

[References	22](#_Toc231752994)

This supplementary material has been provided by the authors to give readers additional information.

# Supplementary Methods

## Study participants, the DEGMAR register, and the BIODEGMAR study

Clinical data, plasma, and cerebrospinal fluid (CSF) samples for the ANGMAR study (*Characterization of Biomarker Footprint in Cerebral Amyloid Angiopathy Patients)*^1^ were obtained within the framework of the DEGMAR register and the BIODEGMAR cohort study^2^.

DEGMAR is a prospective, observational registry that systematically collects sociodemographic, clinical, neuropsychological, and neuroimaging data from patients evaluated at the Cognitive and Behavioural Neurology Unit of Hospital del Mar, a tertiary referral center serving a catchment area of approximately 350,000 inhabitants. The registry was designed to ensure standardized diagnostic evaluation and longitudinal follow-up of individuals with cognitive complaints or cognitive decline. In this unit, the diagnostic protocol includes a comprehensive neurological and neuropsychological assessment. Additional procedures—such as brain MRI and/or biomarker assessments in CSF, plasma, or molecular imaging—are performed according to clinical guidelines or, in selected cases, for research purposes.

Clinical diagnoses are established according to current international criteria and include probable or possible Cerebral Amyloid Angiopathy (CAA) based on the modified Boston criteria (version 2.0)^3^; mild cognitive impairment (MCI)^4^; and probable or possible Alzheimer’s dementia (AD-d)^5^. All DEGMAR participants provide written informed consent. Data are prospectively entered into a secure REDCap database (Vanderbilt University)^6^ and undergo continuous quality control.

BIODEGMAR is the associated biobanking and longitudinal observational study that guarantees harmonized biospecimen acquisition, processing, and storage under standardized operating procedures, and provides a unified infrastructure for biospecimen management, data integration, and traceability across research projects.

The ANGMAR project is a prospective, observational study embedded within the BIODEGMAR protocol. ANGMAR provides the clinical and neuroimaging framework for patient phenotyping, whereas BIODEGMAR ensures standardized biospecimen handling, quality control, and long-term storage across participants.

The ANGMAR study enrolled eligible patients from the inpatient Neurology Service, including the Neurovascular Unit, as well as from the Cognitive and Behavioural Neurology Unit (memory-clinic outpatients). All cases were prospectively assessed under the same standardized clinical, neuropsychological, MRI, and CSF workflows established by the BIODEGMAR protocol. Plasma and CSF samples were obtained and processed following identical biobanking procedures, ensuring harmonized pre-analytical conditions across recruitment sources.

## BIODEGMAR protocol and procedures

*Clinical evaluation:* All participants underwent a structured neurological assessment performed by certified neurologists. The evaluation included detailed medical history, neurological examination, and clinical diagnosis according to established international criteria. Information on vascular risk factors, comorbidities, and medication use was systematically collected during the baseline visit and updated at follow-up.

*Neuropsychological evaluation:* Performed by trained neuropsychologists following a standardized protocol, including: Mini Mental State Examination^7^, Memory Impairment Screen^8^, Automatic reverse series (Subtest of Test Barcelona cognitive battery, Peña-Casanova, 2005)^9^, Semantic fluency task (Subtest of Test Barcelona cognitive battery, Peña-Casanova, 2005)^9^, Free and Cued Selective Reminding Test^10^, Boston Naming Test^11^, Trail Making Test^12^, Blessed Dementia Rating Scale^13^, Alzheimer’s Disease Functional Assessment and Change Scale^14^, Global Deterioration Scale (GDS)^15^, and Clinical Dementia Rating (CDR) Scale^16^.

*Neuroimaging:* MRI scans were acquired on a 3T scanner (Philips Achieva) using harmonized acquisition parameters. The imaging protocol included T1 and T2 weighted sequences, high-resolution 3D T1, diffusion-weighted imaging (DWI), fluid-attenuated inversion recovery (FLAIR), T2-weighted gradient echo (T2*-GRE), and susceptibility weighted imaging (SWI) sequences, **ensuring full assessment of both structural and hemorrhagic CAA markers. Image rating was performed by experienced neuroradiologists blinded to clinical and biomarker data.**

*APOE genotyping:* performed at the Laboratori de Referència de Catalunya (LRC) using allelic discrimination polymerase chain reaction assays (APOE Real Type®, Progenie Molecular, Valencia, Spain). Two polymorphisms were analyzed: rs7412 (g.8041C>T) and rs429358 (g.7903T>C), defining the ε2, ε3, and ε4 alleles that encode the ApoE2, ApoE3, and ApoE4 isoforms, respectively. Both ε4 and ε2 carrier status were considered for analyses: ε4-carriers were defined as individuals with one or two ε4 alleles, ε2-carriers as those with one or two ε2 alleles, and the remaining participants as non-carriers.

The BIODEGMAR protocol guarantees harmonized procedures for biospecimen acquisition, data recording, and storage across all recruitment sources. Identical standard operating procedures were applied to Neurology outpatients and inpatients, ensuring uniformity of clinical, neuropsychological, imaging, and biomarker workflows. Data and sample traceability were managed via the REDCap^6^ platform, with systematic verification of data completeness and internal consistency prior to analysis.

## CSF and plasma collection and storage

Cerebrospinal fluid (CSF) and plasma samples were collected simultaneously under fasting conditions (at least 8 hours) following the standardized procedures of the BIODEGMAR biobanking protocol. Lumbar puncture was performed at the L3/L4, L4/L5, or L5/S1 intervertebral spaces using a standard spinal needle, between 8:00 and 11:00 a.m. CSF was collected into 10 mL sterile polypropylene tubes (Sarstedt, Nümbrecht, Germany; cat. no. 62.610.201). Immediately after collection, tubes were gently inverted 5–10 times, centrifuged at 2,000 g for 10 minutes at 4 °C, and the supernatant was aliquoted in 1.8 mL volumes into sterile polypropylene cryotubes (Thermo Scientific™ Nunc™, Thermo Fisher Scientific, Waltham, MA, USA; cat. no. 377267). All aliquots were immediately frozen at −80 °C for long-term storage.

Lumbar puncture was not performed in all eligible participants due to refusal or medical contraindication. In participants with CAA and a history of intracerebral hemorrhage (ICH) or subarachnoid hemorrhage (SAH), the procedure was delayed by at least three months after the acute event to minimize potential confounding effects of acute-phase CSF alterations.

Whole blood was drawn on the same day using a 20- or 21-gauge needle into 10 mL K₂-EDTA tubes (BD Vacutainer™, Becton Dickinson, USA; cat. no. 367525). Tubes were gently inverted 5–10 times and centrifuged at 2,000 g for 10 minutes at 4 °C. The plasma supernatant was aliquoted in 1.8 mL volumes into the same type of sterile polypropylene cryotubes used for CSF and immediately frozen at −80 °C.

## AD CSF core biomarker measurements and cutoff values

Core AD CSF biomarkers (Aβ42, Aβ40, p-tau181 and t-tau) were quantified at the Laboratori de Referència de Catalunya (LRC, Barcelona, Spain) using the fully automated Lumipulse G600II platform (Fujirebio^17^). After collection and preanalytical processing according to the BIODEGMAR standardized protocol, CSF samples were aliquoted into 0.5 mL sterile polypropylene tubes (Sarstedt, Nümbrecht, Germany; cat. no. 72.730.006) at the Hospital del Mar laboratory and immediately frozen on dry ice for shipment to the LRC, where they were stored at −80 °C until analysis.

CSF biomarker cutoffs were defined in the CORCOBIA study^2^, which established reference thresholds for core AD biomarkers across two centers (Parc de Salut Mar, Barcelona, and Hospital General de Granollers), both operating under the same analytical standards as LRC. Biomarker concentrations were compared between cognitively unimpaired individuals (n = 42) and patients with mild AD dementia (n = 48). Optimal cutoffs were determined using the Youden index (sensitivity + specificity − 1).

The resulting cutoffs and their diagnostic performance (area under the curve, AUC) were as follows: Aβ42, 750 pg/ml (AUC = 0.81); Aβ42/Aβ40, 0.062 (AUC = 0.78); p-tau181, 69.85 pg/ml (AUC = 0.81); t-tau, 522.0 pg/ml (AUC = 0.79); Aβ42/p-tau181, 10.25 (AUC = 0.86).

A full description of the CORCOBIA study design, analytical procedures, and validation of cutoffs is provided in Puig-Pijoan et al.^2^.

## Plasma p-tau217 measurements and cutoff values

Plasma phosphorylated tau at threonine 217 (p-tau217) concentrations were quantified using the Lumipulse pTau217 Plasma assay on fully automated Lumipulse analyzers (G600II or G1200; Fujirebio). Analyses were performed in two certified laboratories—the Laboratori de Referència de Catalunya (LRC) and the Barcelonaβeta Brain Research Center (BBRC)—following standardized preanalytical and analytical procedures within the BIODEGMAR framework. All samples underwent identical preanalytical processing at Hospital del Mar and its laboratory of reference (LRC) before biomarker determination. Analyses were performed using the same analytical methodology and harmonized procedures, including internal and external quality controls.

The Lumipulse pTau 217 Plasma assay is a two-step chemiluminescent immunoassay in which the analyte is first captured on RD85-coated particles in the presence of an assay-specific solution and, after washing, detected with an alkaline-phosphatase–labeled HT7/BT2 conjugate. Quantification is based on a synthetic peptide containing the three assay-specific epitopes as calibrator. Internal and external quality-control materials were included in every analytical run, and inter- and intra-assay coefficients of variation remained below 6 %.

The analytical validation and diagnostic performance of this assay were established in Palmqvist et al. (2025)^18^, in which the BIODEGMAR cohort participated. That multicenter study included 1,767 individuals with cognitive symptoms, recruited from four secondary-care cohorts—Malmö (Sweden, n = 337), Gothenburg (Sweden, n = 165), Barcelona (Spain, n = 487), and Brescia (Italy, n = 230)—as well as a primary-care cohort in Sweden (n = 548). Plasma p-tau217 was quantified using the same automated Lumipulse immunoassay. Alzheimer’s disease pathology was defined by an abnormal CSF Aβ42/p-tau181 ratio (<11.94).

In the Malmö discovery cohort, a single plasma cutoff of >0.27 pg/mL, corresponding to 90% specificity, identified AD pathology with an accuracy of 89% (95% CI 86–93%) for identifying AD pathology. Applying this threshold to independent secondary-care cohorts yielded similar accuracies: 91% (95% CI 86–95%) in Gothenburg, 89% (95% CI 86–92%) in Barcelona, and 89 % (95% CI 85–93%) in Brescia.

To further stratify risk, a two-cutoff approach was defined in the discovery cohort, corresponding to 95% sensitivity and 95% specificity (upper cutoff >0.34 pg/ml, lower cutoff <0.22 pg/ml). This method achieved an accuracy of 93% (95% CI 90–96%) in the same cohort with 17% of participants falling within the intermediate zone between both thresholds. When replicated across secondary-care cohorts, accuracies were 94% (95% CI 90–98%) in Gothenburg, 94 % (95% CI 92–96%) in Barcelona, and 92% (95% CI 88–96 %) in Brescia, with 12–14% of participants in the intermediate range. Across cohorts, positive and negative predictive values (PPV/NPV) were ≥90 %, except for the NPV in Brescia (84 %, 95 % CI 75–92 %), consistent with the higher prevalence of AD pathology (71%) in that sample.

In the present study, plasma p-tau217 was measured using the same Lumipulse pTau217 Plasma assay on fully automated Lumipulse analyzers. The thresholds of 0.22, 0.27, and 0.34 pg/mL were used as reference values for descriptive and comparative analyses.

## Protocol approvals, registrations, and participant consent

The ANGMAR study and the BIODEGMAR cohort were approved by the local ethics committee (CEIm Hospital del Mar Research Institute; project codes 2021/9960/I and 2018/7805/I, respectively). Participants enrolled in the BIODEGMAR cohort could undergo lumbar puncture either for clinical purposes—according to current diagnostic guidelines and recommendations^19^—or exclusively for research purposes. In each case, specific informed consent was obtained. All participants, or their legal representatives when applicable, provided written informed consent in accordance with the Declaration of Helsinki.

# Supplementary Results

## Clinical and radiological profile of CAA subgroups

Within the CAA subgroup, clinical manifestations refer to the symptoms experienced before inclusion in the registry and MRI acquisition. The overall frequency of cognitive impairment at presentation did not differ significantly between CAA+AD**−** and CAA+AD+. However, when the course of cognitive decline was considered, progressive deterioration — as reported by the patient or an informant and confirmed by the clinician — was significantly more frequent in the CAA+AD+ subgroup (**Supplementary Table S1**). The distribution of other presenting symptoms, such as intracerebral haemorrhage or transient focal neurological episodes, was comparable between groups (all p > 0.10). Consistent with this, individuals with CAA+AD+ showed more advanced cognitive and functional impairment than those with CAA+AD**−**, reflected by lower MMSE scores and higher CDR global stages (p < 0.05; **Supplementary Table S1).**

## Plasma p-tau217 association with demographic, clinical, and genetic variables

Associations between plasma p-tau217 concentrations and demographic, clinical, and genetic factors are summarized in **Supplementary Figure S3** and **Supplementary Table S3**. Median p-tau217 values by group and univariate test statistics are displayed for sex, hypertension, diabetes, dyslipidemia, and *APOE* ε2/ε4 carriership.

# Supplementary Figures

## Figure S1. Study flowchart

Participants were recruited from the Neurology Service at Hospital del Mar, including memory-clinic outpatients (Cognitive and Behavioural Neurology Unit) and Neurology inpatients (including the Neurovascular Unit). All participants followed the same diagnostic workflow (ANGMAR/BIODEGMAR). Abbreviations: HC, healthy controls; CAA, cerebral amyloid angiopathy; AD, Alzheimer’s disease; CSF, cerebrospinal fluid; MCI, mild cognitive impairment; NIA-AA, National Institute on Aging–Alzheimer’s Association; A+T+, positive amyloid and tau CSF biomarkers.

## Figure S2. Distribution of p-tau217 in the ANGMAR cohort

Histograms represent the distribution of p-tau217 concentration with and without log-transformation.

## Figure S3. Clinical correlates of plasma p-tau217

Violin plots depicting the distribution of plasma p-tau217 across demographic and clinical variables. Group differences were assessed using Mann–Whitney U tests, and corresponding p-values are shown.

## Figure S4. Distribution of log p-tau217 across diagnostic groups

Density functions of p-tau217 coloured by diagnostic groups.

## Figure S5. Differences in plasma p-tau217 by possible and probable CAA

Distribution of p-tau217 across diagnostic groups with possible and probable CAA. Differences between the possible and probable groups were assessed using the Mann–Whitney U test.

*Key*: ns, not significant.

## Figure S6. Differences in plasma p-tau217 by groups excluding possible CAA

Sensitivity analysis excluding cases with possible CAA. **(A)** Marginal means from an ANCOVA model examining differences in log-transformed plasma p-tau217 across diagnostic groups, adjusted for age and sex. **(B)** Pairwise comparisons derived from the ANCOVA model. Blue tiles indicate comparisons that remained significant after Bonferroni correction. For each comparison, Cohen’s |*d*| and corresponding p-values are shown.

## Figure S7. Differences in plasma p-tau217 by accumulation of MRI CAA markers

Marginal means from an ANCOVA model showing differences in log-transformed plasma p-tau217 according to the CAA–SVD burden score, adjusted for age and sex.

## Figure S8. Differences in plasma p-tau217 by individual MRI CAA markers

Marginal means from ANCOVA models showing differences in log-transformed plasma p-tau217 according to each MRI marker of CAA, adjusted for age and sex.

# Supplementary Tables

## Table S1. CAA participants’ clinical and radiological characteristics

| **Clinical manifestations** | **CAA (n=50)** | **CAA+AD−**  **(n=21)** | **CAA+AD+**  **(n=29)** | ***p*-value** |
| --- | --- | --- | --- | --- |
| Lobar ICH (*n*, %) | 10 (20.0%) | 7 (33.3%) | 3 (10.3%) | 0.073 |
| SAH (*n*, %) | 2 (4.00%) | 1 (4.76%) | 1 (3.45%) | 1.000 |
| TFNEs (*n*, %) | 7 (14.0%) | 4 (19.0%) | 3 (10.3%) | 0.434 |
| Cognitive impairment (*n*, %) | 40 (80.0%) | 15 (71.4%) | 25 (86.2%) | 0.286 |
| Progressive decline (*n*, %) | 29 (58.0%) | 7 (33.3%) | 22 (75.9%) | 0.015 |
| MMSE | 23.0 [18.2;26.8] | 25.5 [22.5;28.0] | 20.5 [17.2;24.0] | 0.018 |
| CDR |  |  |  | 0.020 |
| 0 | 8 (16.0%) | 7 (33.3%) | 1 (3.45%) |  |
| 0.5 | 16 (32.0%) | 7 (33.3%) | 9 (31.0%) |  |
| 1 | 2 (4.00%) | 1 (4.76%) | 1 (3.45%) |  |
| 2 | 19 (38.0%) | 4 (19.0%) | 15 (51.7%) |  |
| 3 | 5 (10.0%) | 2 (9.52%) | 3 (10.3%) |  |
| *APOE* ε4 carriers, *n* (%) | 23 (50.0%) | 3 (15.8%) | 20 (74.1%) | <0.001 |
| *APOE* ε2 carriers, *n* (%) | 8 (17.4%) | 5 (26.3%) | 3 (11.1%) | 0.246 |
| **Radiological characteristics** |  |  |  |  |
| Lobar CMBs (*n*, %) |  |  |  | 0.211 |
| 0 | 3 (6.00%) | 2 (9.52%) | 1 (3.45%) |  |
| 1-4 | 26 (52.0%) | 10 (47.6%) | 16 (55.2%) |  |
| 5-10 | 5 (10.0%) | 4 (19.0%) | 1 (3.45%) |  |
| >10 | 16 (32.0%) | 5 (23.8%) | 11 (37.9%) |  |
| cSS (*n*, %) | 18 (36.0%) | 10 (47.6%) | 8 (27.6%) | 0.247 |
| CSO severe PVS (*n*, %) | 11 (22.0%) | 7 (33.3%) | 4 (13.8%) | 0.166 |
| Fazekas score |  |  |  | 0.714 |
| 0 (*n*, %) | 7 (14.0%) | 2 (9.52%) | 5 (17.2%) |  |
| 1 (*n*, %) | 12 (24.0%) | 6 (28.6%) | 6 (20.7%) |  |
| ≥2 (*n*, %) | 31 (62.0%) | 13 (61.9%) | 18 (62.1%) |  |
| **CAA-SVD Burden Score** |  |  |  | 0.915 |
| 0-1 (*n*, %) | 19 (38.0%) | 7 (33.3%) | 12 (41.4%) |  |
| 2 (*n*, %) | 10 (20.0%) | 4 (19.0%) | 6 (20.7%) |  |
| 3 (*n*, %) | 7 (14.0%) | 3 (14.3%) | 4 (13.8%) |  |
| ≥4 (*n*, %) | 14 (28.0%) | 7 (33.3%) | 7 (24.1%) |  |
| **Boston criteria v2.0** |  |  |  | 0.99 |
| Possible CAA (*n*, %) | 12 (24.0%) | 5 (26.8) | 7 (24.1%) |  |
| Probable CAA (*n*, %) | 38 (76.0%) | 16 (76.2%) | 22 (75.9%) |  |

Data are expressed as median and interquartile range or percentage (%).

*Note*: Clinical manifestations refer to symptoms that the patient experienced before inclusion in the registry and brain MRI acquisition. The number of CMBs was quantified using the T2-GRE MRI sequence. Abbreviations: ICH, intracerebral haemorrhage; CMB, cerebral microbleeds; cSS, cortical superficial siderosis; CSO, centrum semiovale; PVS, perivascular spaces; SVD, small-vessel disease; SAH, subarachnoid haemorrhage; TNFEs, transient focal neurologic episodes.

## Table S2. Main characteristics of patients with CAA stratified by possible and probable diagnoses and presence of AD

| **Characteristics** | **CAA+Ps AD− (N=5)** | **CAA+Ps AD+ (N=7)** | **CAA+Pr AD− (N=16)** | **CAA+Pr AD+ (N=22)** | ***p*-value** |
| --- | --- | --- | --- | --- | --- |
| Age, years | 72.0 [64.0;75.0] | 76.0 [73.5;78.5] | 70.0 [66.0;75.0] | 73.5 [70.2;76.0] | 0.174 |
| Sex, n (%) |  |  |  |  | 0.178 |
| Female | 4 (80.0%) | 5 (71.4%) | 5 (31.2%) | 12 (54.5%) |  |
| Male | 1 (20.0%) | 2 (28.6%) | 11 (68.8%) | 10 (45.5%) |  |
| Ethnicity, n (%) |  |  |  |  | 0.551 |
| Caucasian | 4 (80.0%) | 7 (100%) | 15 (93.8%) | 21 (95.5%) |  |
| Others | 1 (20.0%) | 0 (0.00%) | 1 (6.25%) | 1 (4.55%) |  |
| Education, years | 10.0 [8.00;12.0] | 8.00 [7.00;10.0] | 8.00 [8.00;12.0] | 8.00 [6.00;12.0] | 0.559 |
| Hypertension, n (%) | 4 (80.0%) | 6 (85.7%) | 8 (50.0%) | 13 (59.1%) | 0.393 |
| Diabetes Mellitus, n (%) | 1 (20.0%) | 3 (42.9%) | 1 (6.25%) | 4 (18.2%) | 0.165 |
| Dyslipidemia, n (%) | 1 (20.0%) | 4 (57.1%) | 7 (43.8%) | 14 (66.7%) | 0.219 |
| Obesity, n (%) | 1 (20.0%) | 1 (16.7%) | 4 (28.6%) | 1 (5.56%) | 0.238 |
| Ischemic heart disease, n (%) | 0 (0.00%) | 1 (14.3%) | 0 (0.00%) | 1 (4.55%) | 0.524 |
| Antiplatelet therapy, n (%) | 1 (20.0%) | 3 (42.9%) | 2 (12.5%) | 6 (27.3%) | 0.462 |
| MMSE | 26.5 [20.2;30.0] | 24.0 [20.0;25.5] | 25.5 [22.5;28.0] | 20.0 [17.0;23.0] | 0.054 |
| CDR |  |  |  |  | 0.048 |
| 0 | 1 (20.0%) | 0 (0.00%) | 6 (37.5%) | 1 (4.55%) |  |
| 0.5 | 3 (60.0%) | 3 (42.9%) | 4 (25.0%) | 6 (27.3%) |  |
| 1 | 0 (0.00%) | 1 (14.3%) | 1 (6.25%) | 0 (0.00%) |  |
| 2 | 0 (0.00%) | 3 (42.9%) | 4 (25.0%) | 12 (54.5%) |  |
| 3 | 1 (20.0%) | 0 (0.00%) | 1 (6.25%) | 3 (13.6%) |  |
| Fazekas score |  |  |  |  | 0.016 |
| 0 (n, %) | 1 (20.0%) | 2 (28.6%) | 1 (6.25%) | 3 (13.6%) |  |
| 1 (n, %) | 4 (80.0%) | 2 (28.6%) | 2 (12.5%) | 4 (18.2%) |  |
| ≥2 (n, %) | 0 (0.00%) | 3 (42.9%) | 13 (81.2%) | 15 (68.2%) |  |
| *APOE* ε4 carriers, n (%) | 0 (0.00%) | 4 (66.7%) | 3 (20.0%) | 16 (76.2%) | 0.001 |
| *APOE* ε2 carriers, n (%) | 1 (25.0%) | 0 (0.00%) | 4 (26.7%) | 3 (14.3%) | 0.482 |

Data are presented as median (interquartile range) or percentage (%), as appropriate. Group differences were assessed using the Kruskal–Wallis test for continuous variables and the 𝜒^2^ test for categorical variables.

Missing data: obesity status was unavailable for 7 participants, MMSE for 4 participants, and *APOE* genotype for 4 participants.

## Table S3. Univariate associations between plasma p-tau217 concentrations and demographic and clinical variables

| **Variable** | **Statistic** | **p-value** | **Test Type** |
| --- | --- | --- | --- |
| Age | 0.09 | 0.15472 | Correlation Spearman |
| Sex | 2.33 | 0.12673 | Kruskal-Wallis Test |
| Education | -0.15 | 0.02478 | Correlation Spearman |
| Ethnicity | 0.88 | 0.34944 | Kruskal-Wallis Test |
| Hypertension | 1.60 | 0.20589 | Kruskal-Wallis Test |
| Diabetes | 0.46 | 0.49736 | Kruskal-Wallis Test |
| Dyslipidemia | 4.07 | 0.04362 | Kruskal-Wallis Test |
| Obesity | 0.36 | 0.54919 | Kruskal-Wallis Test |
| Previous ischemic cardiopathy | 0.02 | 0.88886 | Kruskal-Wallis Test |
| Use of antiplatelet drugs | 0.00 | 0.98093 | Kruskal-Wallis Test |
| *APOE* ε2 | 2.74 | 0.09773 | Kruskal-Wallis Test |
| *APOE* ε4 | 12.53 | 0.00040 | Kruskal-Wallis Test |

Associations were assessed using Spearman rank correlation for continuous variables and Kruskal–Wallis test for categorical variables. The table presents test statistics (ρ or χ²) and corresponding p-values.

## Table S4. Descriptive statistics of plasma and CSF biomarkers by diagnostic group

| **Biomarker** | **All**  **(n = 231)** | **HC**  **(*n* = 27)** | **CAA+AD−**  **(n=21)** | **CAA+AD+**  **(n=29)** | **CAA−AD+**  **(n=154)** | ***p*-value** |
| --- | --- | --- | --- | --- | --- | --- |
| Plasma p-tau217 (pg/mL) | 0.57 [0.32;0.87] | 0.10 [0.08;0.16] | 0.19 [0.14;0.29] | 0.59 [0.48;0.94] | 0.68 [0.52;1.00] | <0.001 |
| CSF Aβ42 (pg/mL) | 597 [460;765] | 1254 [920;1520] | 596 [381;1092] | 395 [331;484] | 598 [498;717] | <0.001 |
| CSF Aβ40 (pg/mL) | 13144 [10476;15925] | 14035 [11294;16222] | 9075 [7740;12482] | 10376 [9217;12552] | 14109 [11510;16810] | <0.001 |
| CSF p-tau181 (pg/mL) | 108 [73.8;142] | 42.6 [34.4;54.2] | 45.6 [35.6;55.8] | 118 [89.0;134] | 123 [98.8;160] | <0.001 |
| CSF t-tau (pg/mL) | 670 [478;891] | 356 [256;403] | 313 [243;369] | 725 [595;991] | 740 [598;984] | <0.001 |
| CSF Aβ42/Aβ40 ratio | 0.04 [0.04;0.05] | 0.10 [0.08;0.11] | 0.06 [0.05;0.09] | 0.04 [0.03;0.04] | 0.04 [0.04;0.05] | <0.001 |
| CSF Aβ42/p-tau181 ratio | 5.11 [3.71;7.71] | 31.2 [24.7;40.0] | 18.3 [8.25;25.2] | 3.51 [2.72;4.30] | 4.81 [3.43;5.99] | <0.001 |

Data are expressed as median [interquartile range]. Comparisons across diagnostic groups (HC, CAA+AD**−**, CAA+AD+, and CAA**−**AD+) were performed using Kruskal–Wallis tests. Plasma p-tau217 and CSF biomarkers were quantified using Lumipulse automated assays. Significant group differences (p < 0.001 for all biomarkers) are shown in the rightmost column.

## Table S5. Descriptive statistics of plasma and CSF biomarkers of patients with CAA stratified by possible and probable diagnoses and presence of AD

| **Biomarker** | **CAA+Ps AD− (N=5)** | **CAA+Ps AD+ (N=7)** | **CAA+Pr AD− (N=16)** | **CAA+Pr AD+ (N=22)** | ***p*-value** |
| --- | --- | --- | --- | --- | --- |
| Plasma p-tau217 (pg/mL) | 0.13 [0.09;0.41] | 0.59 [0.54;1.06] | 0.20 [0.15;0.27] | 0.61 [0.47;0.78] | <0.001 |
| CSF Aβ42 (pg/mL) | 1172 [984;1390] | 418 [347;572] | 532 [361;728] | 393 [334;429] | 0.006 |
| CSF Aβ40 (pg/mL) | 11085 [9075;13298] | 10591 [9756;12345] | 8402 [6528;11818] | 10162 [8912;12263] | 0.168 |
| CSF p-tau181 (pg/mL) | 44.3 [36.8;54.1] | 116 [97.2;125] | 46.8 [34.9;56.2] | 118 [86.4;141] | <0.001 |
| CSF t-tau (pg/mL) | 314 [277;335] | 606 [600;688] | 308 [237;382] | 767 [596;996] | <0.001 |
| CSF Aβ42/Aβ40 ratio | 0.10 [0.10;0.11] | 0.04 [0.03;0.05] | 0.06 [0.05;0.09] | 0.04 [0.03;0.04] | <0.001 |
| CSF Aβ42/p-tau181 ratio | 34.6 [26.5;37.8] | 3.51 [2.90;4.19] | 12.1 [7.74;22.9] | 3.41 [2.78;4.28] | <0.001 |

Data are expressed as median [interquartile range]. Comparisons across diagnostic groups (HC, CAA+AD−, CAA+AD+, and CAA−AD+) were performed using Kruskal–Wallis tests. Plasma p-tau217 and CSF biomarkers were quantified using Lumipulse automated assays. Significant group differences are shown in the rightmost column.

# References

1.	Fernández‐Lebrero A, Jiménez‐Balado J, García‐Escobar G, et al. CSF Biomarker Profile of Cerebral Amyloid Angiopathy: Diagnostic Performance and Imaging Correlates in a Hospital‐Based Neurology Cohort. *Eur J Neurol*. 2026;33(5):e70613. doi:10.1111/ene.70613

2.	Puig-Pijoan A, García-Escobar G, Fernández-Lebrero A, et al. The CORCOBIA study: Cut-off points of Alzheimer’s disease CSF biomarkers in a clinical cohort. *Neurol Engl Ed*. 2024;39(9):756-765. doi:10.1016/j.nrleng.2022.05.002

3.	Charidimou A, Boulouis G, Frosch MP, et al. The Boston criteria version 2.0 for cerebral amyloid angiopathy: a multicentre, retrospective, MRI–neuropathology diagnostic accuracy study. *Lancet Neurol*. 2022;21(8):714-725. doi:10.1016/S1474-4422(22)00208-3

4.	Albert MS, DeKosky ST, Dickson D, et al. The diagnosis of mild cognitive impairment due to Alzheimer’s disease: Recommendations from the National Institute on Aging‐Alzheimer’s Association workgroups on diagnostic guidelines for Alzheimer’s disease. *Alzheimers Dement*. 2011;7(3):270-279. doi:10.1016/j.jalz.2011.03.008

5.	McKhann GM, Knopman DS, Chertkow H, et al. The diagnosis of dementia due to Alzheimer’s disease: Recommendations from the National Institute on Aging‐Alzheimer’s Association workgroups on diagnostic guidelines for Alzheimer’s disease. *Alzheimers Dement*. 2011;7(3):263-269. doi:10.1016/j.jalz.2011.03.005

6.	Harris PA, Taylor R, Thielke R, Payne J, Gonzalez N, Conde JG. Research electronic data capture (REDCap)—A metadata-driven methodology and workflow process for providing translational research informatics support. *J Biomed Inform*. 2009;42(2):377-381. doi:10.1016/j.jbi.2008.08.010

7.	Folstein MF, Folstein SE, McHugh PR. “Mini-mental state.” *J Psychiatr Res*. 1975;12(3):189-198. doi:10.1016/0022-3956(75)90026-6

8.	Buschke H, Kuslansky G, Katz M, et al. Screening for dementia with the Memory Impairment Screen. *Neurology*. 1999;52(2):231-231. doi:10.1212/WNL.52.2.231

9.	Peña-Casanova J. *Test Barcelona Revisado: programa integrado de exploración neuropsicológica : TBR*. 2a ed. Masson; 2005.

10.	Buschke H. Cued recall in Amnesia. *J Clin Neuropsychol*. 1984;6(4):433-440. doi:10.1080/01688638408401233

11.	Roth C. Boston Naming Test. In: Kreutzer JS, DeLuca J, Caplan B, eds. *Encyclopedia of Clinical Neuropsychology*. Springer New York; 2011:430-433. doi:10.1007/978-0-387-79948-3_869

12.	Reed JC, Reed HBC. The Halstead—Reitan Neuropsychological Battery. In: Goldstein G, Incagnoli TM, eds. *Contemporary Approaches to Neuropsychological Assessment*. Critical Issues in Neuropsychology. Springer US; 1997:93-129. doi:10.1007/978-1-4757-9820-3_4

13.	Blessed G, Tomlinson BE, Roth M. The Association Between Quantitative Measures of Dementia and of Senile Change in the Cerebral Grey Matter of Elderly Subjects. *Br J Psychiatry*. 1968;114(512):797-811. doi:10.1192/bjp.114.512.797

14.	Galasko D, Bennett D, Sano M, et al. An Inventory to Assess Activities of Daily Living for Clinical Trials in Alzheimerʼs Disease: *Alzheimer Dis Assoc Disord*. 1997;11:33-39. doi:10.1097/00002093-199700112-00005

15.	The Global Deterioration Scale for assessment of primary degenerative dementia. *Am J Psychiatry*. 1982;139(9):1136-1139. doi:10.1176/ajp.139.9.1136

16.	Morris JC. The Clinical Dementia Rating (CDR): Current version and scoring rules. *Neurology*. 1993;43(11):2412. doi:10.1212/WNL.43.11.2412-a

17.	Gobom J, Parnetti L, Rosa-Neto P, et al. Validation of the LUMIPULSE automated immunoassay for the measurement of core AD biomarkers in cerebrospinal fluid. *Clin Chem Lab Med CCLM*. 2022;60(2):207-219. doi:10.1515/cclm-2021-0651

18.	Palmqvist S, Warmenhoven N, Anastasi F, et al. Plasma phospho-tau217 for Alzheimer’s disease diagnosis in primary and secondary care using a fully automated platform. *Nat Med*. Published online April 9, 2025. doi:10.1038/s41591-025-03622-w

19.	*Guía oficial de práctica clínica en demencia*. Luzán 5 Health Consulting S.A. ; Ediciones SEN; 2018.
